# 3′-tRF-Cys^GCA^ overexpression in HEK-293 cells alters the global expression profile and modulates cellular processes and pathways

**DOI:** 10.1007/s10142-023-01272-0

**Published:** 2023-11-21

**Authors:** Paraskevi Karousi, Martina Samiotaki, Manousos Makridakis, Jerome Zoidakis, Diamantis C. Sideris, Andreas Scorilas, Thomas Carell, Christos K. Kontos

**Affiliations:** 1 Department of Biochemistry and Molecular Biology, Faculty of Biology, National and Kapodistrian University of Athens, Panepistimiopolis, 15701 Athens, Greece; 2 Institute for Bioinnovation, Biomedical Sciences Research Center, “Alexander Fleming”, Vari, Greece; 3 Center of Systems Biology, Biomedical Research Foundation of the Academy of Athens, Athens, Greece; 4 Department for Chemistry, Institute for Chemical Epigenetics, Ludwig Maximilian University of Munich, Munich, Germany

**Keywords:** tRNA-derived RNA fragment (tRF), Non-coding RNA, Post-transcriptional regulation, Transcriptomics, Proteomics

## Abstract

tRNA fragments (tRFs) are small non-coding RNAs generated through specific cleavage of tRNAs and involved in various biological processes. Among the different types of tRFs, the 3′-tRFs have attracted scientific interest due to their regulatory role in gene expression. In this study, we investigated the role of 3′-tRF-Cys^GCA^, a tRF deriving from cleavage in the T-loop of tRNA^CysGCA^, in the regulation of gene expression in HEK-293 cells. Previous studies have shown that 3′-tRF-Cys^GCA^ is incorporated into the RISC complex and interacts with Argonaute proteins, suggesting its involvement in the regulation of gene expression. However, the general role and effect of the deregulation of 3′-tRF-Cys^GCA^ levels in human cells have not been investigated so far. To fill this gap, we stably overexpressed 3′-tRF-Cys^GCA^ in HEK-293 cells and performed transcriptomic and proteomic analyses. Moreover, we validated the interaction of this tRF with putative targets, the levels of which were found to be affected by 3′-tRF-Cys^GCA^ overexpression. Lastly, we investigated the implication of 3′-tRF-Cys^GCA^ in various pathways using extensive bioinformatics analysis. Our results indicate that 3′-tRF-Cys^GCA^ overexpression led to changes in the global gene expression profile of HEK-293 cells and that multiple cellular pathways were affected by the deregulation of the levels of this tRF. Additionally, we demonstrated that 3′-tRF-Cys^GCA^ directly interacts with thymopoietin (*TMPO*) transcript variant 1 (also known as *LAP2α*), leading to modulation of its levels. In conclusion, our findings suggest that 3′-tRF-Cys^GCA^ plays a significant role in gene expression regulation and highlight the importance of this tRF in cellular processes.

**Supplementary Information:**

The online version contains supplementary material available at 10.1007/s10142-023-01272-0.

## Introduction

Transfer RNA (tRNA) fragments (tRFs) are small non-coding RNA molecules that are produced by the cleavage of tRNA molecules. Various endoribonucleases, exoribonucleases, and specific aminoacyl-tRNA synthetases can trigger the cleavage of tRNA, resulting in the formation of tRFs (Lee et al. 2009). The biogenesis of tRFs can occur under various conditions such as cellular stress, viral infections, and cell differentiation (Kumar et al. 2016; Martinez 2018). Several classes of tRFs have been described based on their size and the cleavage site on the tRNA molecule from which they derive. The cleavage sites can be located in distinct parts of the tRNA molecule; depending on their origin, tRFs can be classified into several types, including tRNA-derived small RNAs (tsRNAs) and tRNA halves or tRNA-derived stress-induced RNAs (tiRNAs). The most frequently investigated tRFs are 5′-tRFs and 3′-tRFs, which are generated from the 5′ and 3′ ends of mature tRNAs, respectively, while tRFs deriving from the internal loop of the tRNA (i-tRFs) also exist (Li et al. 2018; Shen et al. 2018).

tRFs have been implicated in different biological processes, including translation regulation, RNA interference, DNA damage response, cell proliferation, apoptosis, and various other processes (Goodarzi et al. 2016; Saikia et al. 2014; Telonis et al. 2019; Zhang et al. 2020). The 5′- and 3′-tRF classes have aroused scientific interest utmost, due to their implication in various of the aforementioned processes, and mostly in the regulation of gene expression. Specifically, these tRFs have been shown to target the 3′-untranslated region (3′-UTR) of mRNAs, leading to either stabilization or destabilization of the target transcript depending on the specific tRF and mRNA combination (Green et al. 2020; Guan et al. 2020; Kumar et al. 2014); even more interestingly, this function is executed through the RISC complex (Kuscu et al. 2018).

In addition to post-transcriptional regulation, 3′-tRFs are implicated in the regulation of translation. Specifically, some 3′-tRFs are able to bind to the ribosomal A-site, which is the location on the ribosome where the tRNA anticodon binds to the mRNA codon during translation. This binding inhibits translation initiation by preventing the binding of the initiator tRNA to the ribosomal A-site (Kim et al. 2017; Luo et al. 2018). Furthermore, it has been suggested that 3′-tRFs may interact with the translation initiation factors eIF4G and eIF4A to regulate translation (Gebetsberger and Polacek 2013), while it has also been reported that 3′-tRFs upregulate the expression of ribosomal protein mRNAs by binding to the 5′-UTR of these mRNAs, which enhances their translation (Kumar et al. 2015).

In this work, we chose to investigate the role of a fragment deriving from cleavage in the T-loop of one of the tRNAs bearing the cysteine anticodon GCA (tRNA^CysGCA^). This fragment, which will be mentioned as 3′-tRF-Cys^GCA^, has been reported to be incorporated in the RISC complex and specifically to interact with AGO1 and AGO2 (Green et al. 2020; Kuscu et al. 2018). In this framework, this fragment has been proven to interact with mRNAs by complementary base-pairing and affect their stability (Green et al. 2020; Kuscu et al. 2018; Xiao et al. 2021). However, the general role and effect of the deregulation of the levels of 3′-tRF-Cys^GCA^ in human cells has not been investigated so far. Therefore, we stably overexpressed 3′-tRF-Cys^GCA^ in HEK-293 and performed transcriptomics and proteomics experiments to explore the effect of 3′-tRF-Cys^GCA^ in the expression profile of the cells. Extensive bioinformatics analysis revealed the implication of this tRF in various pathways, while luciferase reporter assays provided evidence for the interaction of 3′-tRF-Cys^GCA^ with mRNAs of particular genes, the expression levels of which were found to be modulated upon 3′-tRF-Cys^GCA^ overexpression.

## Methodology

### Cell culture and DNA isolation

The HEK-293 embryonic kidney cell line was cultivated following the guidelines of ATCC®. Next, total DNA was isolated after lysis of the cells in TRI Reagent® (Molecular Research Center, Inc. Cincinnati, OH, USA) and following the manufacturer’s instructions. The concentration and purity of the isolated DNA were assessed spectrophotometrically in a BioSpec-nano Micro-volume UV–Vis Spectrophotometer (Shimadzu, Kyoto, Japan).

### tRF selection

We chose to investigate the role of 3′-tRF-Cys^GCA^, a fragment of the mature tRNA-Cys^GCA^, with the following sequence: 5′-UCCGGGUGCCCCCUCCA-3′. This 3′-tRF is recorded in two public repositories, namely tRFdb (ID: tRF-3003a) and MINTbase v2.0 (ID: tRF-17-8871K92) (Kumar et al. 2015; Pliatsika et al. 2018, 2016) and may derive from ten distinct tRNA^CysGCA^ genes: *TRC-GCA2-1*, *TRC-GCA2-2*, *TRC-GCA2-3*, *TRC-GCA2-4*, *TRC-GCA4-1*, *TRC-GCA5-1*, *TRC-GCA7-1*, *TRC-GCA8-1*, *TRC-GCA11-1*, and *TRC-GCA14-1*.

This 3′-tRF was selected among others for several reasons; firstly, it has been reported to interact with AGO proteins (Kumar et al. 2014), thus rendering it possible to have a regulatory role. Additionally, it is one of the most abundant tRFs, as it has been detected in various small RNA sequencing (RNA-seq), with high reads per million (RPM) values, as shown in tRFdb (Kumar et al. 2015). Additionally, this tRF is one of the two most abundant 3′-tRFs deriving from the aforementioned tRNAs, as shown in the MINTbase v2.0 data and presented in Fig. S1. Lastly, overexpression of *TRC-GCA2-4* (also known as *chr17.trna25*), a tRNA gene located on chr17:39154491–39154562 (-), has already been shown to lead to the overexpression of the aforementioned 3′-tRF (Kuscu et al. 2018).

### Primer designing and PCR

One primer pair was designed to amplify a gene region that included the *TRC-GCA2-4* (tRNA^CysGCA^) gene and its flanking sequences (200 nucleotides before its 5′ end and 192 nucleotides after its 3′ end). The coordinates of the amplified region are chr17:39154299–39154762 (-) (*Homo sapiens* genome assembly GRCh38.p14 (hg38)). The sense and antisense primers were designed to contain the recognition sequences of the restriction endonucleases SacI and XbaI, respectively. The total DNA isolated from the HEK-293 cell line was used as a template for PCR. The primer pair and the annealing temperature are shown in Table S1. More details about the reaction mixture and the thermal protocol can be found in Supplementary Methodology.

### Agarose gel electrophoresis, gel clean-up, and Sanger sequencing

The PCR product generated through the amplification of the aforementioned genomic region was electrophorized in a 2% agarose gel. After visualization in UV light, the band was excised, and DNA was extracted using spin columns (Macherey–Nagel GmbH & Co. KG, Düren, Germany). The concentration of the extracted PCR product was evaluated using a Qubit 2.0 Fluorometer (Invitrogen™, Thermo Fisher Scientific Inc., Carlsbad, CA, USA). Subsequently, the sequence was confirmed by Sanger sequencing.

### Plasmid construction, bacteria cell transformation, and plasmid purification

The TOPO™ TA Cloning™ kit (Invitrogen™) was used to ligate the pCR™II-TOPO™ vector with the purified PCR product, following the manufacturer’s guidelines. The ligation mixture was used to transform competent *E. coli* cells. After transformation, bacteria were spread in Luria Broth (LB) agar selection plates. Next, colony selection was done by blue-white screening. The selected colonies were inoculated in liquid bacteria cell culture. Plasmid purification followed, using spin columns (Macherey–Nagel GmbH & Co. KG, Düren, Germany). Next, the recombinant pCR™II-TOPO™ vector and the PCMV6-Neo (OriGene, Rockville, MD, USA) backbone were digested using SacI and XbaI restriction enzymes (New England Biolabs Ltd., Hitchin, UK). The restricted products were electrophoresed, and both the linearized PCMV6-Neo backbone and the insert for the recombinant pCR™II-TOPO™ vector were cleaned up using spin columns (Macherey–Nagel GmbH & Co. KG, Düren, Germany). The ligation procedure was repeated to ligate the linearized PCMV6-Neo backbone and the PCR product of the *TRC-GCA2-4* gene and its flanking sequences. After the construction and clean-up of the recombinant PCMV6-Neo vector, the SmaI restriction enzyme (New England Biolabs Ltd.) was used to linearize the recombinant PCMV6-Neo vector. More details about the reaction mixtures and the thermal protocols can be found in Supplementary Methodology.

### Cell transfection and clone selection

To transfect HEK-293 cells, 10^5^ cells were seeded in two wells of a 24-well plate. Lipofectamine 2000 (Invitrogen™) was used to transfect 500 ng of the recombinant linearized PCMV6-Neo vector. To transfect cells, 2 µL of Lipofectamine® 2000 reagent (Invitrogen™) were diluted in 50 µL of Opti-MEM® medium (Invitrogen™). In parallel, 500 ng of the linear recombinant plasmid were diluted in 250 µL of Opti-MEM® medium. The two solutions were then mixed in a 1:1 ratio. Then, the DNA-lipofectamine mixture was incubated at room temperature for 5 min, and 50 µL of the mixture was added to the one well, while the other one served as negative control. Forty-eight (48) hours after transfection, the medium was replaced by a medium containing 600 µg/mL geneticin (G418; AppliChem GmbH, Darmstadt, Germany), to select those cells that had been transfected with the vector. The antibiotic concentration had previously been determined by an antibiotic kill curve, ranging from 0.2 to 0.8 mg/mL G418. After 14 days of selection, each cell that had incorporated the vector was transfected in a well of a 96-well plate. In this way, 3 HEK-293 clones were developed.

### Nucleic acid extraction and PCR

Total DNA and RNA were extracted from the HEK-293 parental cell line and the three clones using the TRI Reagent® (Molecular Research Center, Inc.). To ensure the repeatability of the results, two genomic DNA and two total RNA extracts were isolated from each clone, while two genomic DNA and three total RNA extracts were isolated from the parental cell line. After determining the concentration and purity of the nucleic acids spectrophotometrically, the integrity of the RNA extracts was assessed after electrophoresis in a Bioanalyzer 2100 RNA nano chip (Agilent Technologies, Winooski, VT, USA). Next, the DNA extracts were used as template to conduct a PCR assay, to ensure the incorporation of the recombinant vector in the HEK-293 clone genome. The primer annealing temperature is shown in Table S1. More details about the reaction mixture and the thermal protocol can be found in Supplementary Methodology.

### In vitro polyadenylation, cDNA synthesis, and real-time quantitative PCR (qPCR)

After ensuring the incorporation of the recombinant plasmid in the HEK-293 genome, each total RNA extract was subjected to in vitro polyadenylation, as previously described (Karousi et al. 2021). Next, the polyadenylated RNA extract was used as a template to conduct first-strand cDNA synthesis, using MML-V reverse transcriptase (Invitrogen™) and an oligo-dT adapter primer, following the manufacturer’s instructions, with the only exception being that the denaturation step was performed at 30 °C; we performed the denaturation at such low temperature in order to ensure that only tRFs and not tRNAs would be denatured and reversely transcribed. The oligo-dT adapter primer sequence was 5′-GCGAGCACAGAATTAATACGACTCACTATAGGTTTTTTTTTTTTVN-3′.

Specific forward primers were designed for 3′-tRF-Cys^GCA^, *SNORD43* and *SNORD61* (C/D box-containing small nucleolar RNAs 43 and 61, respectively), which served as reference genes for 3′-tRF-Cys^GCA^ normalization. The reverse primers were complementary to the oligo-dT adaptor sequence used during reverse transcription (Table S1). Real-time qPCR assays were developed and optimized. For this purpose, a standard curve was generated for each amplicon, using serial cDNA dilutions. The expression levels of 3′-tRF-Cys^GCA^ were calculated using the comparative C_T_ (2^−∆∆CT^) method (Livak and Schmittgen 2001; Schmittgen and Livak 2008). More details about the reaction mixtures and the thermal protocols can be found in Supplementary Methodology.

### Poly(A)-RNA selection, library construction, and RNA-seq

Oligo-dT-based magnetic mRNA isolation from 5 µg of each RNA extract (two of each clone and three of the parental cell line) was conducted using NEBNext® Poly(A) mRNA Magnetic Isolation Module (New England Biolabs Ltd.). Each poly(A) enriched extract was used to generate a barcoded sequencing library, using the MGIEasy RNA Directional Library Prep Set (MGI Tech Co. Ltd., Shenzhen, China) and following the manufacturer’s instructions. RNA-seq was conducted in the DNBSEQ-G50 platform (MGI Tech Co. Ltd.), using a DNBSEQ-G50RS High-throughput Sequencing Set providing paired-end 100 chemistry.

### Quantitative proteomics using data-independent acquisition (DIA)

Three biological replicates of 10^6^ cells of each clone and the parental cell line were pelleted after washing with PBS and subjected to filter-aided sample preparation (FASP), in order to generate tryptic peptides (Wisniewski et al. 2009). The peptides were cleaned up according to the Sp3 strategy (Hughes et al. 2019), using a 1:1 mix of Sera-Mag™ SpeedBead Carboxylate-Modified [E3] and [E7] Magnetic Particles (Cytiva, Marlborough, MA, USA).

Equal amounts of peptide mixtures were analyzed by liquid chromatography with tandem mass spectrometry (LC–MS/MS) in two technical replicates for each of the three biological replicates. LC–MS/MS analysis was performed by injecting the peptidic eluate directly onto an analytical column (25 cm × 75 µm, 1.9 µm beads, C18 ReproSil AQ, Bruker GmbH, Mannheim, Germany), followed by gradient elution in an UltiMate™ 3000 RSLCnano system (Thermo Scientific™, Thermo Fisher Scientific Inc., Waltham, MA, USA). The separated peptides were ionized and sprayed into the Q Exactive™ HF-X Mass Spectrometer (Thermo Scientific™) through a PepSep Stainless Steel Emitter with Liquid Junction (Bruker GmbH). The mass spectrometer was operated in data-independent acquisition (DIA) mode, followed by data-independent analysis.

### Data post-processing, biostatistics, and bioinformatics

Post-processing and bioinformatics analysis of the RNA-seq data was conducted using the Partek® Flow software (Partek Inc., Chesterfield, MO, USA). Specifically, quality control and trimming of low-quality bases were conducted, prior to the alignment of the reads; the reads were aligned to the human genome 38 (GRCh38) using the RNA STAR aligner (Dobin et al. 2013). In order to quantify gene expression, each read was assigned to one of the RefSeq transcripts and normalized. Differential expression analysis between the three clones and the parental cell line was conducted with the gene-specific analysis (GSA) module of Partek® Flow. The metrics of the RNA-seq experiment are shown in Table S2.

Regarding proteomics experiments, the Orbitrap raw data were analyzed in DIA-NN 1.8.1 (Data-Independent Acquisition by Neural Networks) (Demichev et al. 2020), through searching against the Human Proteome (downloaded from UniProt as one protein per gene, 20,583 protein entries, 11/2022) using the library free mode of the software, allowing up to two tryptic missed cleavages and a maximum of three variable modifications/peptide. The search was used with oxidation of methionine residues, N-terminal methionine excision, and acetylation of the protein N-termini set as variable modifications and carbamidomethylation of cysteine residues as fixed modification. A spectral library was created from the DIA runs and used to reanalyze them (double search mode). The match between runs feature was used for all analyses, and the output (precursor) was filtered at 0.01 FDR, and finally, the protein inference was performed on the level of genes using only proteotypic peptides.

The statistical analysis was performed within Perseus (v.1.6.15.0) (Tyanova et al. 2016). The four groups (clone 1, clone 2, clone 3, and parental cell line) were filtered for at least 70% valid (detected) values in at least one of the groups. The remaining missing values were imputed based on the Gaussian distribution. A two-sample Welch’s *t*-test with a *P* value of less than 0.05 was performed. The results obtained from these comparisons resulted in three lists, representing the differentially expressed genes between each clone and the parental cell line. The intersection (common proteins) between these three lists was used to create the final differentially expressed protein list. Enrichment analysis for both transcriptomics and proteomics data was conducted using the differentially expressed RNA and protein lists as input in Metascape (https://metascape.org) (Zhou et al. 2019).

Putative 3′-tRF-Cys^GCA^ targets were retrieved from the tRFtar, tRFtarget, tRFtars, and tRForest databases (Li et al. 2021; Parikh et al. 2022; Xiao et al. 2021; Zhou et al. 2021). The union of four target lists resulted in the final putative target list. The list of the putative targets, the differentially expressed RNAs list, and the differentially expressed protein list were compared and integrated.

### Determination of cellular growth rate

To assess the proliferation rates of both parental and clonal cells, a fundamental technique involving cell counting in a hemacytometer chamber was utilized. In this routine procedure, each potential clone or control cell population was initially seeded in a 24-well plate at an initial concentration of 1 × 10^5^ cells. At specific time intervals of 24, 48, 72, and 96 h, the cells were subjected to trypsinization, followed by washing, resuspension in trypan blue, and subsequent cell counting, using the Countess™ Automated Cell Counter (Invitrogen™).

The sulforhodamine B (SRB) assay was also used as an additional way to ascertain the cell density at every time point. The cells were initially plated into a 96-well plate in triplicate at a concentration of 1 × 10^5^ cells/mL, with each well containing 100 µL of cell suspension. To determine cell density, the SRB assay was employed at four distinct time intervals subsequent to the initial seeding, specifically at 24, 48, 72, and 96 h, as previously described (Karousi et al. 2023). The cell counts and OD values were used to build growth curves for the clonal and parental cells, to assess the growth rate.

### Quantification of putative 3′-tRF-Cys^GCA^ targets

Each total RNA extract was used as a template to conduct first-strand cDNA synthesis, using MML-V reverse transcriptase (Invitrogen™) and the aforementioned oligo-dT adapter primer, following the manufacturer’s instructions.

Specific primers were designed for thymopoietin (*TMPO*) transcript variant 1 (also known as *LAP2α*), endoplasmic reticulum-golgi intermediate compartment 1 (*ERGIC1*), and FTO alpha-ketoglutarate dependent dioxygenase (*FTO*), as well as for beta-2-microglobulin (*B2M*) and hypoxanthine phosphoribosyltransferase 1 (*HPRT1*), which served as reference genes (Table S1). Real-time qPCR assays were developed, as described above, by using the same reaction and thermal protocols. The expression levels of *TMPO* transcript variant 1, *ERGIC1,* and *FTO* were calculated using the comparative C_T_ (2^−∆∆CT^) method (Livak and Schmittgen 2001; Schmittgen and Livak 2008).

### Reporter plasmid construction, bacteria transformation, and plasmid purification

The psiCHECK-2 vector (Promega, Madison, WI, USA) was subjected to digestion using NotI and XhoI restriction enzymes (New England Biolabs Ltd). To do this, 1 µg of the vector was mixed with 20 units of each enzyme in rCutSmart buffer (New England Biolabs Ltd.). The mixture was then incubated at 37 °C for 60 min, followed by heat inactivation at 65 °C for 20 min. Afterwards, the reaction mixture was loaded onto an agarose gel, and the bands containing the linearized plasmid were carefully cut out and purified using the Monarch® DNA Gel Extraction Kit (New England Biolabs Ltd). The restricted plasmid was then ligated with the double-strand DNA sequence of part of the 3′-UTR of each mRNA of interest (Table S3), using the NEBuilder HiFi DNA Assembly Cloning Kit (New England Biolabs Ltd.). After the transformation of the NEB 5-alpha Competent *E. coli* cells (New England Biolabs Ltd.) with the recombinant plasmids, bacteria were spread in LB agar selection plates. Colonies were inoculated in liquid culture, and plasmid purification was conducted with ZymoPURE II Plasmid Midiprep Kit (Zymo Research Europe Gmbh, Freiburg, Germany).

### Dual luciferase reporter assay

To conduct a dual luciferase assay, 9.5 × 10^4^ ΗΕΚ-293 cells were seeded into individual wells of a 96-well plate. After a 24-h incubation period, 50 ng of each plasmid construct was transfected into their respective wells using the jetPRIME® transfection reagent (Polyplus, Illkirch, France). Following a 2-h incubation, 2 pmol of 3′-tRF-Cys^GCA^ mimic were introduced into each well using Lipofectamine RNAiMax (Invitrogen™). The sequence of the mimic was 5′-TCCGGGTGCCCCCTCCA-3′. Additionally, wells were prepared with plasmid constructs and a scrambled sequence for normalization purposes (sequence: 5′-AGCCTCTCGTCGCCCGC-3′), and control wells were set up with only the transfection reagents (mock control). Each reaction was performed in triplicate. Twenty-four (24) hours after the 3′-tRF- Cys^GCA^ transfection, luminescent signal quantification of Renilla and firefly luciferases was carried out. The Dual-Glo® Luciferase Assay System (Promega) was utilized for luminescence measurement, and a Cytation 5 Cell Imaging Multimode Reader (BioTek, Agilent Technologies) was used following the standard protocol. A schematic representation of the workflow followed in this work is shown in Fig. S2.

### Western blot

The protein concentration for each protein extract deriving from clonal and parental cells was determined by Bradford assay. Four protein extracts (20 µg each) from each cell line were used. Glyceraldehyde-3-phosphate dehydrogenase (GAPDH) was used as a control. The selection of GAPDH as a reference protein was based on the fact that the levels of this protein did not show any significant alteration as a result of 3′-tRF-Cys^GCA^ overexpression, in contrast with actin beta (ACTB), which showed variations in the analyzed proteomics data and was hence excluded as an unsuitable reference in this study. In brief, two primary antibodies were added at a 1:1000 dilution: a mouse monoclonal anti-LAP2α and a horseradish peroxidase (HRP)-conjugated monoclonal anti-GAPDH. A secondary HRP-conjugated goat anti-mouse IgG was used to indirectly detect TMPO isoform alpha (LAP2α), whereas GAPDH was directly detected without using any secondary antibody. Detection of each targeted protein (TMPO isoform alpha and GAPDH) was performed by the enhanced chemiluminescence (ECL) detection system. Subsequently, the X-ray films were scanned, and image analysis was conducted.

## Results

### 3′-tRF-Cys^GCA^ levels in the HEK-293 clones and the parental cell line

**Fig. 1:**
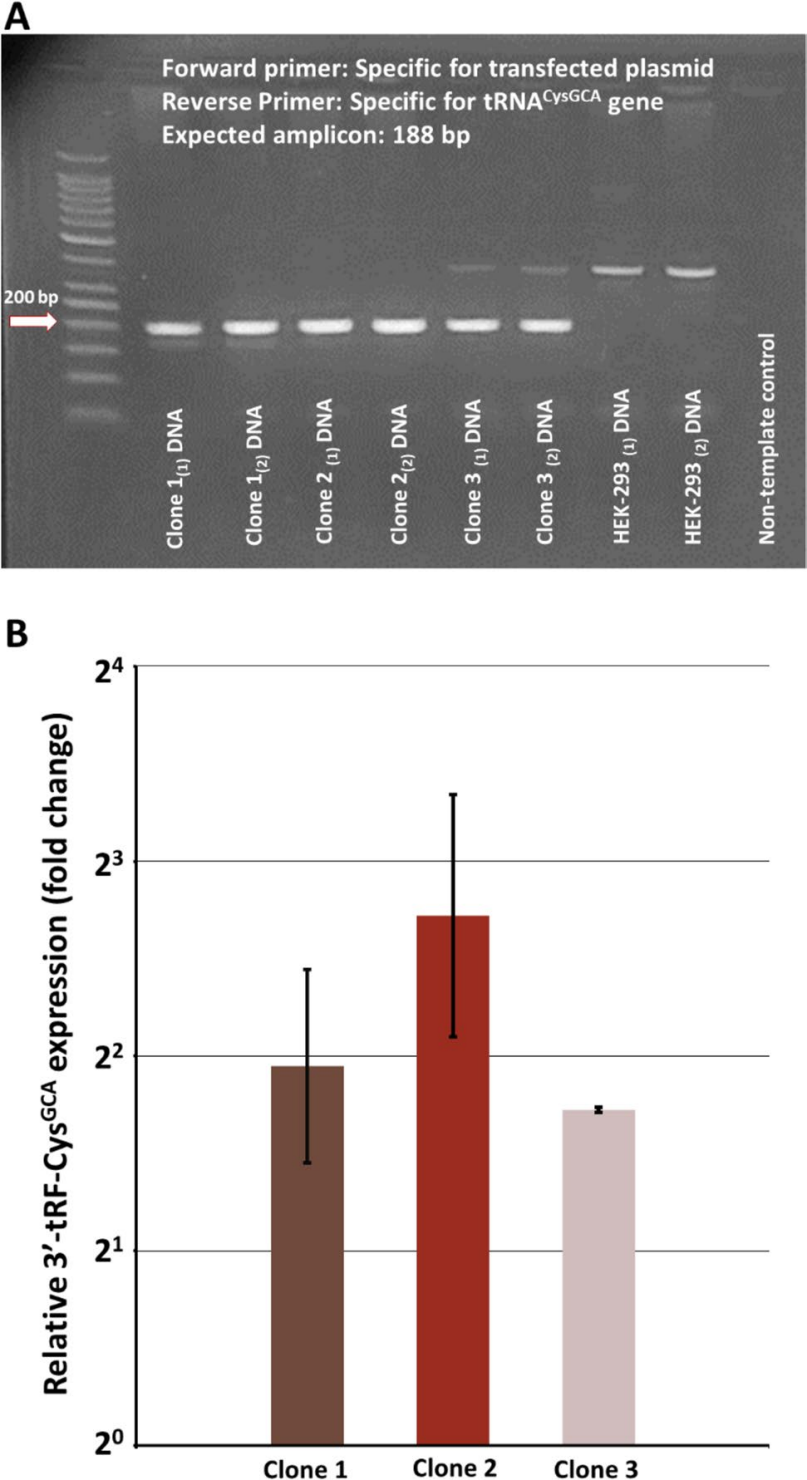
Illustration of the result of 3′-tRF-Cys^GCA^ overexpression. **A** Agarose gel electrophoresis of the PCR products after amplification of the recombinant plasmid in the HEK-293 clone and parental cell line genomes. **B** Normalized expression of 3′-tRF-Cys^GCA^ in the HEK-293 clones. The expression levels of 3′-tRF-Cys^GCA^ were determined using the relative quantification (2^−∆∆Ct^) method: *SNORD43* and *SNORD61* served as endogenous controls, while the cDNAs from HEK-293 were used as calibrator

The successful incorporation was confirmed by assessing the presence of the recombinant vector in DNA extracts from clones and parental cells using PCR. Following the successful incorporation of the recombinant plasmid expressing the *TRC-GCA2-4* (tRNA^CysGCA^) gene into the clone genome (Fig. 1A), we assessed the impact of this genetic modification on 3′-tRF-Cys^GCA^ levels in the resulting clones when compared to the parental HEK-293 cells. As illustrated in Fig. 1B, our findings revealed a significant increase in the relative 3′-tRF-Cys^GCA^ levels in the transfected clones compared to the parental cells. Specifically, clone 1 exhibited a remarkable 3.86-fold elevation in 3′-tRF-Cys^GCA^ levels (with a range of 2.73 – 5.45). Clone 2 displayed an even more pronounced increase, with a 6.60-fold rise in 3′-tRF-Cys^GCA^ levels (ranging from 4.29 to 10.16). Additionally, clone 3 exhibited a notable 3.30-fold enhancement in 3′-tRF-Cys^GCA^ levels (with a range of 3.28–3.34) (Fig. 1B). These findings suggest that the introduction of the recombinant plasmid expressing the *TRC-GCA2-4* gene into the clone genome leads to a substantial upregulation of 3′-tRF-Cys^GCA^ expression.

### Expression profile of the HEK-293 clones and the parental cell line

**Fig. 2:**
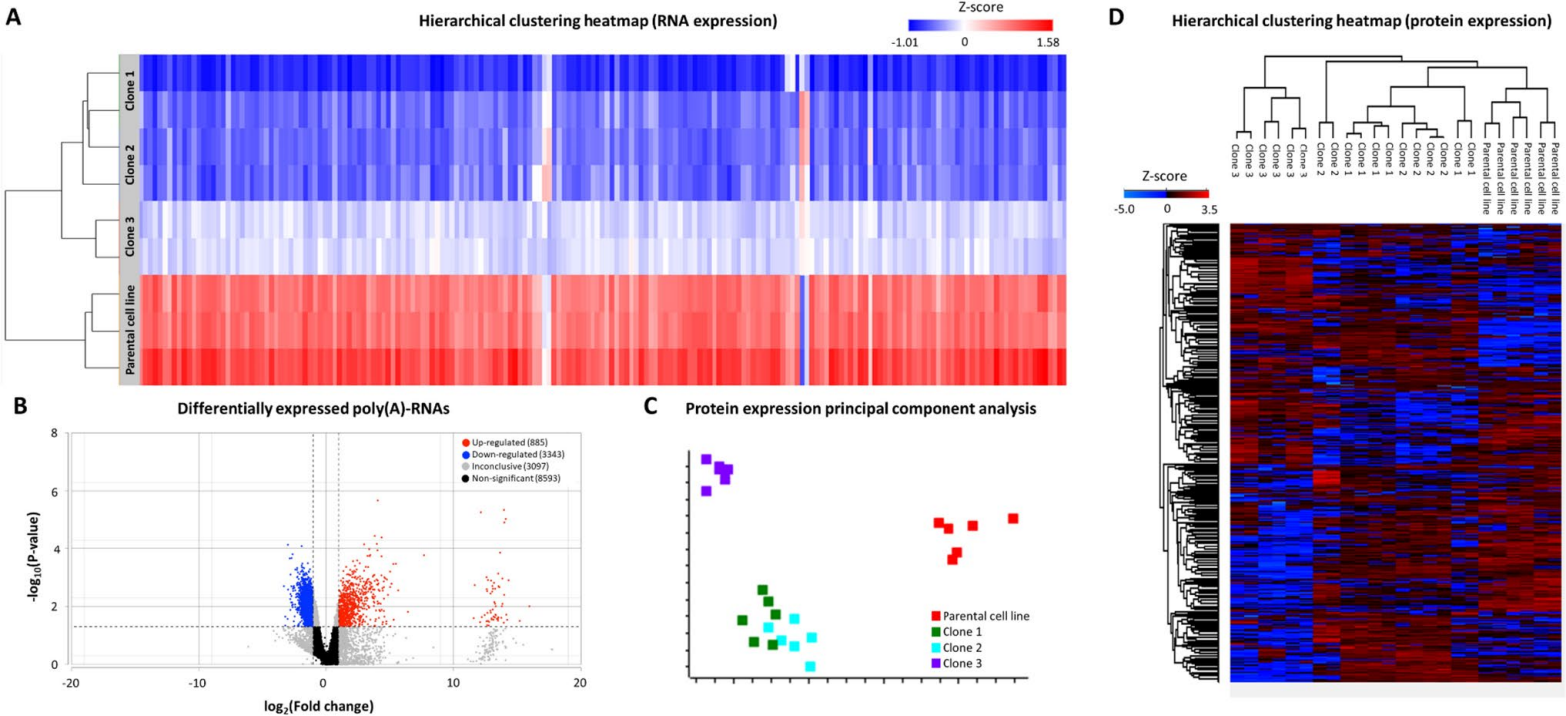
Illustration of the transcriptomics and proteomics experiment results. **A** Hierarchical clustering heatmap, showing the differences in the RNA expression profile of HEK-293 clones *vs* the parental cell line. **B** Volcano plot, showing the differentially expressed RNA in the HEK-293 clones *vs* the parental cell line. **C** Principal component analysis of the protein expression profile of HEK-293 clones and the parental cell line. **D** Hierarchical clustering heatmap, showing the differences in the RNA expression profile of HEK-293 clones *vs* the parental cell line

As depicted in Fig. 2A, the RNA-seq data highlighted significant disparities in the RNA expression profile between the three clones and the parental HEK-293 cells. It is important to note that the replicates within each group complied with each other, ensuring the reliability of our findings. A total of 4228 poly(A)-RNAs exhibited differential expression in the three clones compared to the parental cell line (Table S4). Notably, 3897 of these differentially expressed RNAs originated from protein-coding genes. Even more interestingly, most of the differentially expressed poly(A)-RNAs are downregulated in the clones compared to the parental cell line (Fig. 2B). These findings underscore the profound impact of 3′-tRF-Cys^GCA^ overexpression on the transcriptional landscape.

Concordant with the observed RNA expression profile, the protein expression profile also exhibited significant variations between the three clones overexpressing 3′-tRF-Cys^GCA^ and the parental HEK-293 cells, while the replicates within each group again complied with each other (Fig. 2C, D). Out of the 6742 proteins detected, a substantial subset consisting of 1428 proteins displayed differential expression among the three clones compared to the parental cell line. Detailed information regarding the total number of proteins detected within each biological group and the significant differences in protein levels between each clone and the parental cell line are found in Table S5.

Remarkably, our analysis identified a noteworthy convergence of gene expression changes, with 458 genes exhibiting differential expression at both mRNA and protein levels. This overlap underscores the consistency of the molecular alterations induced by 3′-tRF-Cys^GCA^ overexpression at both transcriptional and translational levels. However, it is important to note that not all 458 common genes displayed uniform regulation patterns at both transcriptional and translational levels. These genes showed complex and nuanced responses, with some being upregulated at the RNA level while downregulated at the protein level, and vice versa.

### Impact of 3′-tRF-Cys^GCA^ overexpression on cellular processes and pathways

**Fig. 3:**
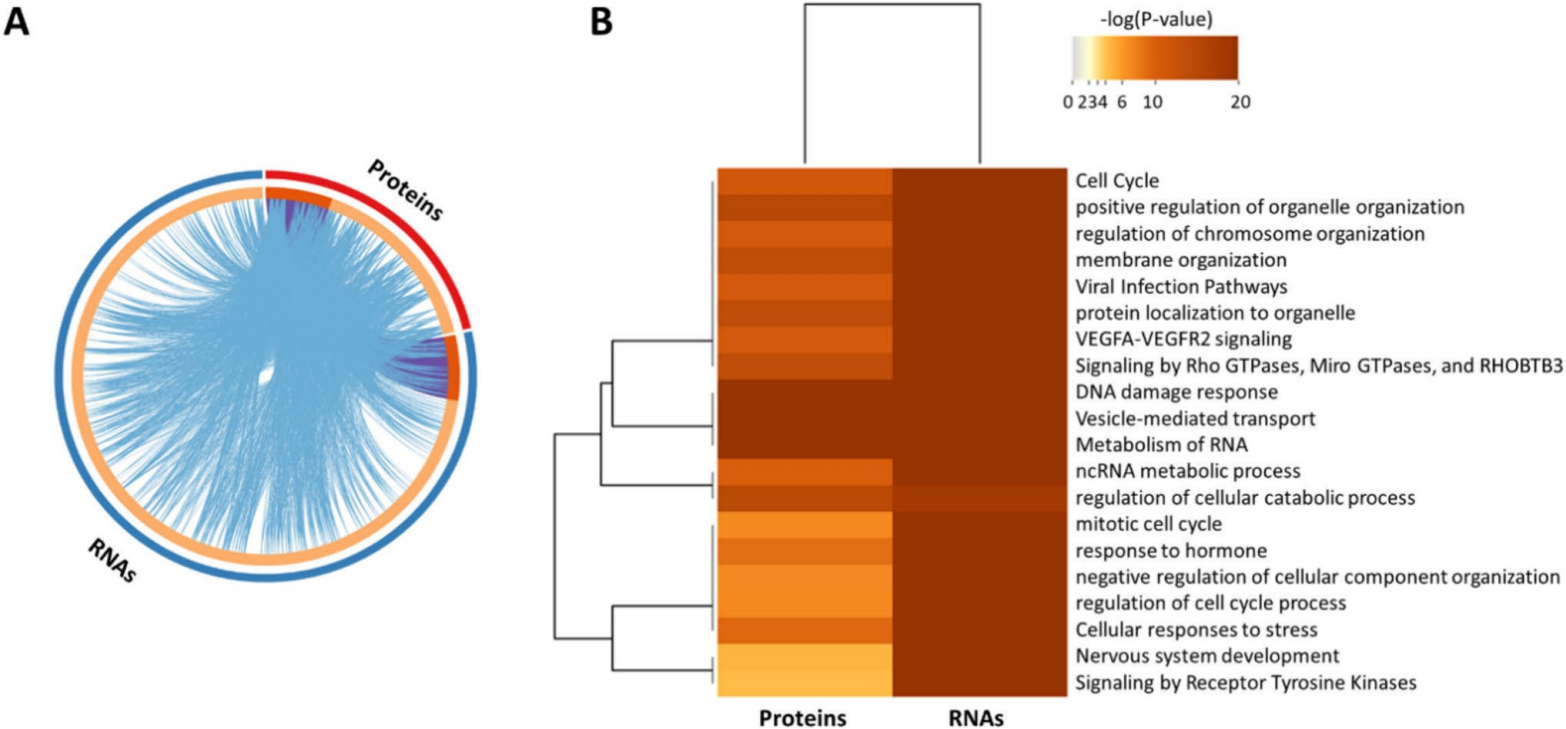
Illustration of the pathway enrichment analysis of the differentially expressed RNAs and proteins. **A** Common genes (purple arc) among the differentially expressed RNAs and proteins, as well as genes that participate in common biological processes and pathways (blue arcs). **B** The biological processes and pathways in which the differentially expressed RNAs and proteins most likely participate

In addition to the 458 genes showing altered expression at both mRNA and protein levels upon 3′-tRF-Cys^GCA^ overexpression, our analysis revealed a multitude of genes that were unique to either the RNA or protein datasets but seemed to participate in the same biological processes (Fig. 3A). This observation suggests a complex regulatory network influenced by the overexpression of 3′-tRF-Cys^GCA^. The overexpression of 3′-tRF-Cys^GCA^ exerted significant alterations in various cellular processes and pathways at both the RNA and protein levels. Pathway analysis highlighted the enrichment of genes associated with the cell cycle pathway. Moreover, positive regulation of organelle organization and regulation of chromosome organization emerged as significantly affected processes. Notably, the overexpressed 3′-tRF-Cys^GCA^ also exhibited an impact on membrane organization and displayed associations with pathways related to viral infection (Fig. 3B). These results reveal an intricate web of regulatory interactions influenced by 3′-tRF-Cys^GCA^, implicating this fragment in the modulation of essential cellular processes and pathways.

**Fig. 4:**
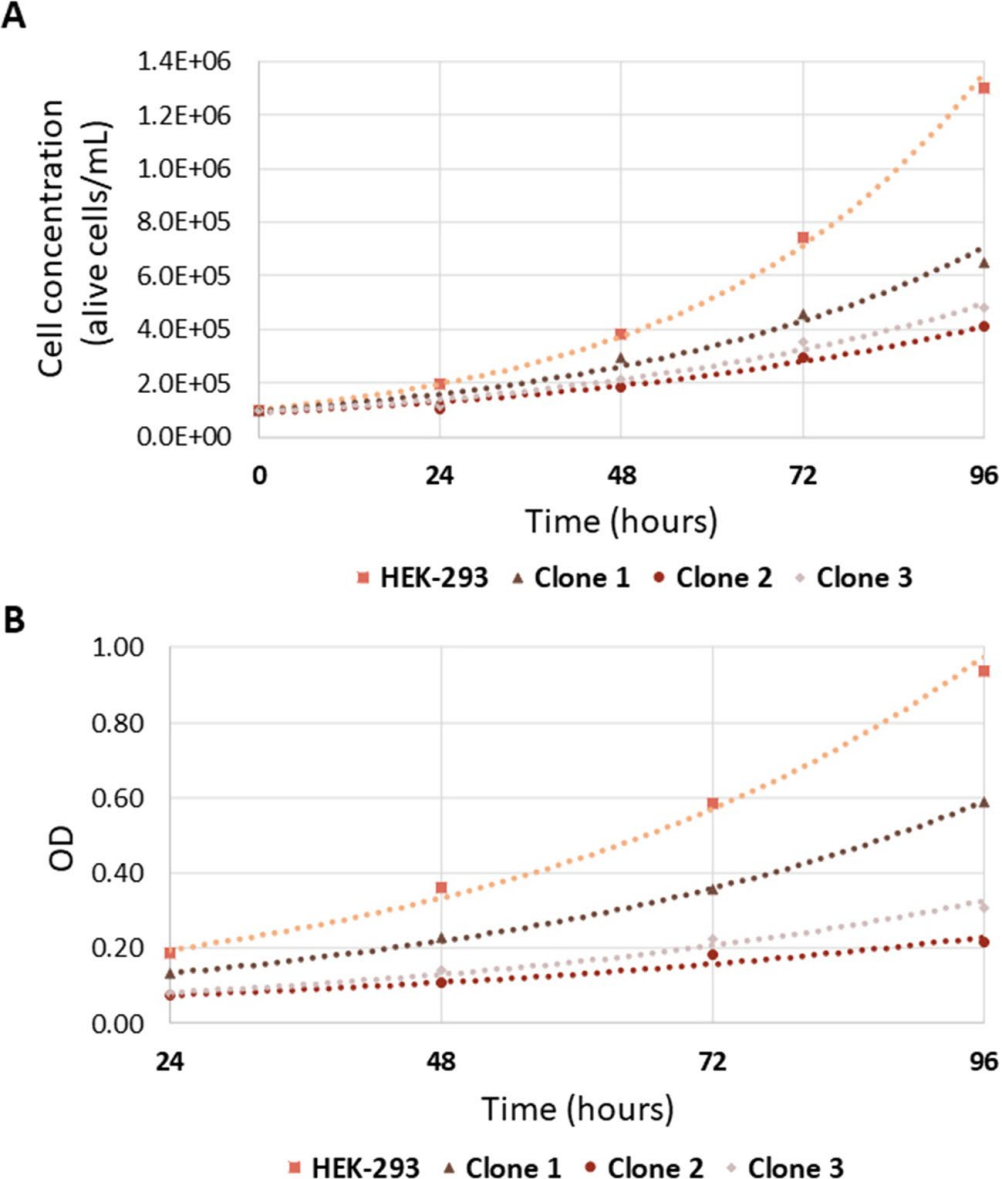
Growth curves of HEK-293 clones and parental cells assessed via trypan blue exclusion (**A**) and SRB assay (**B**). The SRB assay was conducted in triplicate

In order to experimentally evaluate the effect of 3′-tRF-Cys^GCA^ on the cell cycle, we compared the growth rates of HEK-293 parental cells with three distinct HEK-293 clones. During the 96-h observation period, it became evident that all three HEK-293 clones exhibited a noticeable deviation in their proliferation patterns when compared to the parental cells. Among the three distinct HEK-293 clones, it is noteworthy that clones 2 and 3 displayed remarkably similar growth rates, which deviated significantly from the proliferation pattern of the parental cells. Intriguingly, all three clones exhibited a common characteristic—a reduction in proliferation rate compared to the parental HEK-293 cells (Fig. 4).

### Direct interaction of 3′-tRF-Cys^GCA^ with mRNAs

In an effort to validate some of the predicted interactions involving 3′-tRF-Cys^GCA^, we took a systematic approach by generating a putative target list for 3′-tRF-Cys^GCA^. This list was compiled by combining data from four databases specializing in predicting tRF interactions (Table S6). Specifically, the union of the four lists provided us with a list of 969 unique putative targets. To provide further insights, we categorized these putative targets into coding and non-coding and obtained 919 unique putative protein-coding targets. Thirty-five (35) targets were detected in more than one database (Table 1).

**Table 1:** Quantitative data regarding the putative targets of 3′-tRF-Cys^GCA^

Database	Number of putative targets	Number of protein-coding putative targets	Number of unique targets^1^	Number of unique protein-coding targets^1^
tRFTarget	412	402	389	387
tRForest	408	368	380	340
tRFTars	63	61	55	53
tRFTar	121	115	111	104

^1^Unique targets among the four databases

**Fig. 5:**
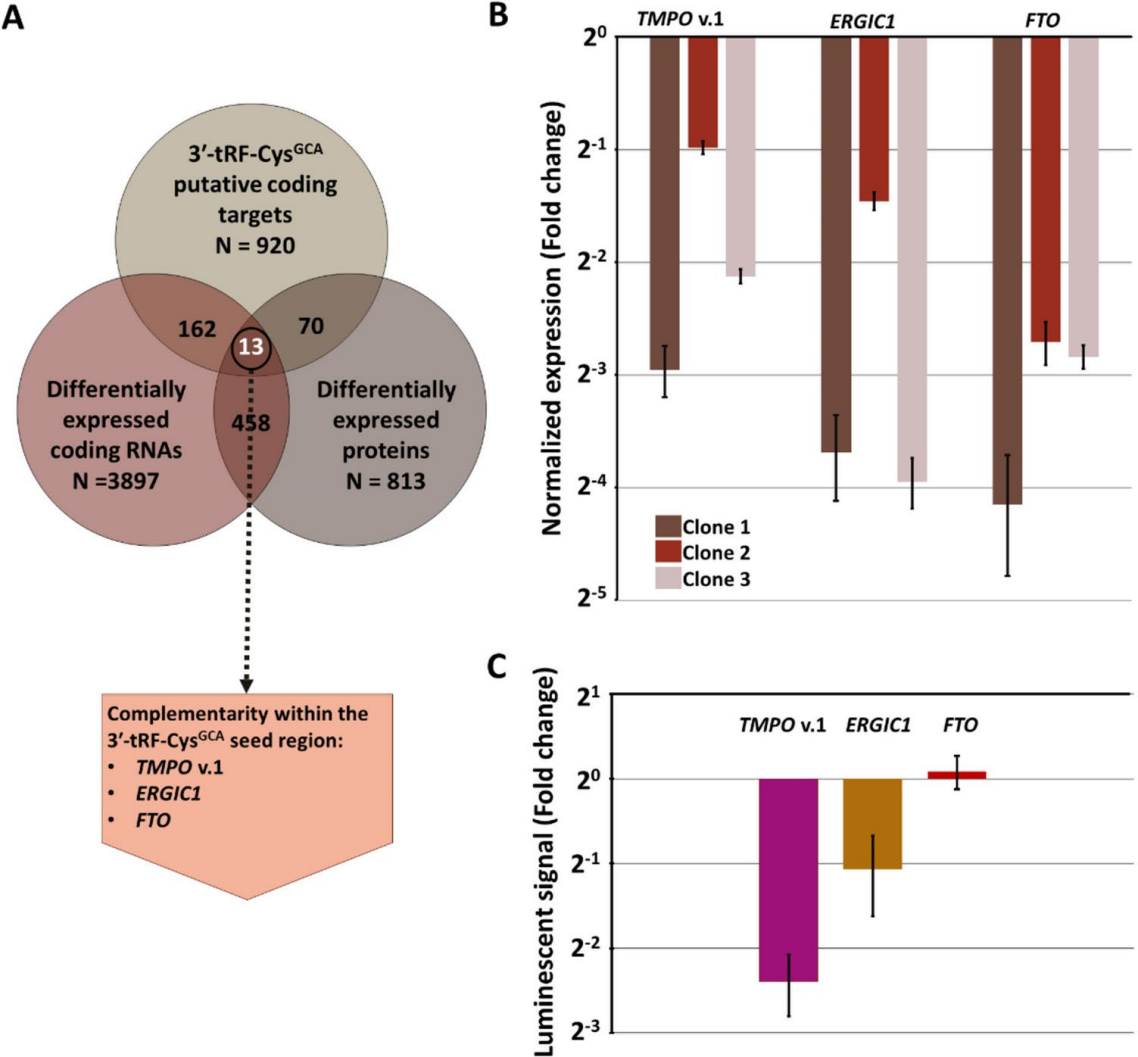
Validation of direct interaction of 3′-tRF-Cys^GCA^ with mRNAs. **A** Venn diagram, showing the common genes among differentially expressed RNAs, differentially expressed proteins and putative 3′-tRF-Cys^GCA^ targets. Among the 13 common genes, three of them are most likely to be direct 3′-tRF-Cys^GCA^ targets. **B** Normalized expression of *TMPO* transcript variant 1 (v.1), *ERGIC1,* and *FTO* in the HEK-293 clones. The expression levels of the aforementioned mRNAs were determined using the relative quantification (2^−∆∆Ct^) method: *HPRT1* and *B2M* served as endogenous controls, while the cDNAs from HEK-293 were used as calibrator. **C** Presentation of the dual luciferase reporter assay results. Normalized ratios were obtained by calculating the *Renilla* to firefly luminescence signal ratio, and then by dividing this ratio with the respective one calculated for the negative control

We then integrated the lists of differentially expressed coding RNAs and proteins with the putative coding target list, identifying 13 genes that were present in all three lists (intersection of the three lists). Among these 13 genes, we first aimed to examine whether one of them is deposited as a putative target of 3′-tRF-Cys^GCA^ in more than one of the aforementioned databases; however, since this query did not provide any results, we selected *TMPO* transcript variant 1, *ERGIC1*, and *FTO* for further validation due to the presence of a short sequence in their 3′-UTR that complements the seed region of 3′-tRF-Cys^GCA^ (Fig. 5A); the respective heteroduplexes between 3′-tRF-Cys^GCA^ and the respective mRNA were confirmed using RNAhybrid (Fig. S3) (Kruger and Rehmsmeier 2006). We did this selection based on a previous study, supporting that complementary of the seed region of 3′-tRF-Cys^GCA^ with the 3′-UTR of mRNAs leads to the downregulation of the latter (Kuscu et al. 2018).

To validate our findings, we initiated the validation process by confirming the reduced mRNA expression levels of *TMPO* transcript variant 1, *ERGIC1*, and *FTO* in the three clones compared to the parental cell line using qPCR (Fig. 5B). Subsequently, a dual luciferase assay was conducted, revealing that 3′-tRF-Cys^GCA^ was capable of repressing the expression of *TMPO* transcript variant 1 (reduction of ~ 80%) and *ERGIC1* (reduction of ~ 50%) but not *FTO*. These results strongly suggest that *TMPO* transcript variant 1is a direct target of 3′-tRF-Cys^GCA^ and lay some possibility for *ERGIC1* being a direct target as well, while *FTO* may not be a direct target (Fig. 5C). Additionally, although Western blot did not show significant differences between the protein levels of TMPO isoform alpha (LAP2α) between the clonal and parental cells (Fig. S4), the impact on *TMPO* gene expression levels is also supported by the LC–MS data, showing reduced TMPO isoform alpha levels in the three clones, compared to the parental cells.

## Discussion

The regulation of gene expression is a complex and tightly controlled process crucial for cellular function and development (Beyersmann 2000). While the canonical role of tRNAs in protein synthesis is well-established, recent studies have uncovered that their derivatives are key players in the intricate landscape of gene expression regulation. tRFs, including 3′-tRFs, possess the ability to influence gene expression at multiple levels, including transcriptional, post-transcriptional, and translational regulation (George et al. 2022). Through their interactions with mRNAs and other regulatory molecules, tRFs have been implicated in modulating diverse biological processes, ranging from cell cycle control and stress responses to development and disease (Bhogireddy et al. 2021; Pandey et al. 2021; Zhang et al. 2020).

The results of our study demonstrate the impact of overexpressing the 3′-tRF-Cys^GCA^ tRNA fragment on various aspects of cellular function and gene expression in HEK-293 cells. Firstly, we observed significantly higher levels of 3′-tRF-Cys^GCA^ in the clones expressing the recombinant plasmid compared to the parental HEK-293 cells. The relative levels of 3′-tRF-Cys^GCA^ were consistently elevated in all three clones, indicating the successful incorporation of the recombinant plasmid into the clone genome. This suggests that the introduced tRNA gene was effectively transcribed and processed, leading to increased levels of 3′-tRF-Cys^GCA^. This fact comes in line with previous studies that have attempted to increase 3′-tRF-Cys^GCA^ levels via tRNA^CysGCA^ gene overexpression (Green et al. 2020; Kuscu et al. 2018; Shen et al. 2018), but also with other studies claiming that tRNA gene overexpression leads to the overexpression of the tRNA derivatives (Torres et al. 2019). In addition to the observed elevation of 3′-tRF-Cys^GCA^ levels, an advantage of the technique used in our study is that the overexpression of the tRNA gene leads to the production of the tRF in conditions very similar to physiological settings. By incorporating the recombinant plasmid expressing the *TRC-GCA2-4* (tRNA^CysGCA^) gene into the clone genome, the tRF is generated through endogenous tRNA processing pathways, ensuring that the tRF is produced in a manner consistent with normal cellular processes. This approach allows for the production of the tRF along with modified bases. o3′-tRF-Cys^GCA^ is mapped to the respective tRNA of origin positions 59 to 75, which are not frequently subjected to modifications (Lorenz et al. 2017).

The RNA expression profile analysis revealed substantial differences between the three clones overexpressing 3′-tRF-Cys^GCA^ and the parental cell line. Numerous poly(A)-RNAs, including a majority derived from protein-coding genes, exhibited differential expression among the clones and the parental cells. This differential expression of poly(A)-RNAs observed between the clones overexpressing 3′-tRF-Cys^GCA^ and the parental cell line may be attributed to various regulatory mechanisms. Firstly, as demonstrated in our study and supported by current literature (Green et al. 2020; Kuscu et al. 2018), the direct interaction of 3′-tRF-Cys^GCA^ with mRNAs can result in translational repression or degradation of specific target transcripts. Furthermore, the altered expression of poly(A)-RNAs could also result indirectly from changes in the expression of transcription factors or other genes involved in the regulation of gene expression. Such an example is the interaction of 3′-tRF-Cys^GCA^ with the *ERGIC1* mRNA and *TMPO* transcript variant 1. *ERGIC1* is an essential component of the endoplasmic reticulum-Golgi intermediate compartment and plays a crucial role in the regulation of vesicular trafficking and protein transport (Breuza et al. 2004). *TMPO* is a gene of special interest since its encoded protein isoforms are known to be involved in the organization of chromatin architecture and acts as transcriptional regulators, influencing gene expression at multiple levels (Dechat et al. 2000; Gant et al. 1999; Mirza et al. 2019). Through the direct interaction with 3′-tRF-Cys^GCA^, *TMPO* transcript variant 1 expression was repressed, as validated in our reporter assay experiments. This repression could have cascading effects on downstream target genes, potentially affecting various cellular processes. Considering the regulatory role of TMPO isoform alpha (LAP2α) in chromatin organization, altered expression levels of *TMPO* gene could influence the accessibility of other gene loci, leading to changes in the expression of genes associated with these regions. This type of indirect regulation, where 3′-tRF-Cys^GCA^ influences the expression of *TMPO* transcript variant 1 (and other mRNAs translated in proteins with similar roles), can induce broader effects on gene expression networks, contributing to the overall changes in the RNA expression profile observed in the clones. Such a hypothesis could also explain the changes in the expression levels of *FTO* and other mRNAs, although they are not direct 3′-tRF-Cys^GCA^ targets.

Consistent with the RNA expression profile, the protein expression profile also differed significantly between the clones and the parental cells. The integration of RNA and protein expression data in our study highlights the complex regulatory mechanisms underlying the effects of 3′-tRF-Cys^GCA^ overexpression. This finding resonates with recent advances in the understanding of post-transcriptional regulation, emphasizing the importance of considering both transcriptional and translational regulation to gain a comprehensive view of gene expression control (Corbett 2018). The aforementioned findings support the notion that tRNA fragments can impact gene expression at multiple levels, further underscoring their functional relevance in cellular processes. tRFs have been shown to modulate translation efficiency and accuracy by interacting with components of the translation machinery. For instance, a study demonstrated that specific tRNA fragments can bind to ribosomes and translation initiation factors, thereby influencing translation initiation and elongation processes (Gonskikh et al. 2020). Even more interestingly, the tiRNAs generated by the 5′ of tRNA^Cys^ inhibit translation as well (Ivanov et al. 2011); such fragments could be generated by the overexpression of tRNA^CysGCA^ and contribute to the altered protein expression profile observed. Moreover, the multiple-level gene expression regulation could explain differences among RNA and protein level alteration in the HEK-293 clones. This observation that not all of the common genes displayed uniform regulatory patterns at both the mRNA and protein levels is in line with findings from previous studies. This phenomenon, where mRNA and protein levels do not necessarily correlate, has been widely documented and underscores the existence of intricate post-transcriptional regulatory mechanisms (Cenik et al. 2015; Edfors et al. 2016).

The altered gene expression patterns and the involvement of various genes in similar biological processes indicate that the overexpressed 3′-tRF-Cys^GCA^ affects multiple cellular pathways. Pathway analysis revealed enrichment in the cell cycle pathway, suggesting a potential regulatory role of the tRNA fragment in cell cycle progression. Previous research has implicated tRNA fragments in the regulation of cell cycle progression, with specific tRNA-derived fragments shown to influence cell cycle checkpoints and cell proliferation in malignancies (Gong et al. 2023). The decelerated proliferation observed in all HEK-293 clones overexpressing 3′-tRF-Cys^GCA^ suggests that this tRNA fragment may function as a negative regulator of the cell cycle, thereby influencing the rate of cell division. Understanding the precise mechanisms by which 3′-tRF-Cys^GCA^ exerts this effect will be essential for elucidating its potential as a target for therapeutic interventions in diseases characterized by aberrant cell cycle progression.

Additionally, processes such as organelle organization, chromosome organization, membrane organization, and viral infection pathways were significantly affected. tRNA fragments have been implicated in various cellular stress responses, including viral infections, where they can modulate antiviral immune responses and viral replication processes (Ivanov 2015). However, further investigation into the mechanisms and pathways through which 3′-tRF-Cys^GCA^ exerts its influence in the aforementioned processes is essential.

In interpreting the results of our study, it is essential to consider limitations that may influence the generalizability and interpretation of our findings. Firstly, the use of a specific cell line (HEK-293) in our experiments may limit the broader applicability of our results to other cell types or tissues. Different cell types exhibit distinct gene expression profiles and regulatory mechanisms, and the responses to 3′-tRF-Cys^GCA^ overexpression might vary among different cell contexts. Secondly, the introduction of a recombinant plasmid for 3′-tRF-Cys^GCA^ overexpression could potentially lead to unintended off-target effects. Changes in cellular pathways or gene expression unrelated to 3′-tRF-Cys^GCA^ may arise due to experimental perturbations. Besides the side effects affecting the clone expression profile, the integration site of 3′-tRF-Cys^GCA^ in the genome could play a critical role in determining its expression levels and processing, which, in turn, can lead to different gene expression profiles. Variability in tRF expression levels among the clones might result from different genomic locations of integration, influencing the availability of the tRF for target binding and subsequent gene expression regulation. To minimize this limitation, we developed three distinct HEK-293 clones, considered biological replicates, while technical replicates were included in all experiments performed in this work. It is essential to note the challenges encountered in reliably quantifying TMPO isoform alpha levels through Western blotting, due to the semi-quantitative nature of this technique. Considering that *TMPO* gene expression levels were found to be altered upon 3′-tRF-Cys^GCA^ overexpression as documented by RNA-seq, qPCR, and LC–MS, and that this is most likely a direct effect as evidenced by our luciferase reporter assay results, we could hypothesize that TMPO isoform alpha expression is moderately modulated by 3′-tRF-Cys^GCA^.

In conclusion, our study demonstrates that overexpression of 3′-tRF-Cys^GCA^ leads to significant alterations in gene expression at both the RNA and protein levels. This tRNA fragment appears to modulate various cellular processes and pathways, potentially influencing organelle dynamics, viral responses, and cell cycle progression. Furthermore, our findings provide evidence of direct interactions between 3′-tRF-Cys^GCA^ and specific mRNAs, leading to repression of target gene expression. Overall, these results contribute to our understanding of the regulatory functions of 3′-tRF-Cys^GCA^ and its involvement in cellular processes, highlighting the complex interplay between this fragment and gene expression regulation. Understanding the roles of tRFs in gene expression regulation provides novel insights into the complex mechanisms governing cellular physiology and holds promise for future therapeutic interventions targeting these regulatory molecules.

### Supplementary Information

Below is the link to the electronic supplementary material.

Supplementary file1 (ZIP 7237 KB)

## Data Availability

The raw sequencing reads have been deposited to the Sequence Read Archive (SRA) of NCBI, with BioProject accession number PRJNA999076. The mass spectrometry proteomics data have been deposited to the ProteomeXchange Consortium via the PRIDE partner repository with the dataset identifier PXD044218.
