# Supplementary material for: 3′-tRF-CysGCA overexpression in HEK-293 cells alters the global expression profile and modulates cellular processes and pathways
Source: Funct Integr Genomics. 2023 Nov 21;23(4):341. doi: 10.1007/s10142-023-01272-0 (PMC10663186; doi:10.1007/s10142-023-01272-0)
Supplement: Supplementary file 1 — Supplementary file1 (ZIP 7237 KB) [file 10142_2023_1272_MOESM1_ESM.zip › Supplementary Material/Supplementary Figures.docx]

**Supplementary Figures**


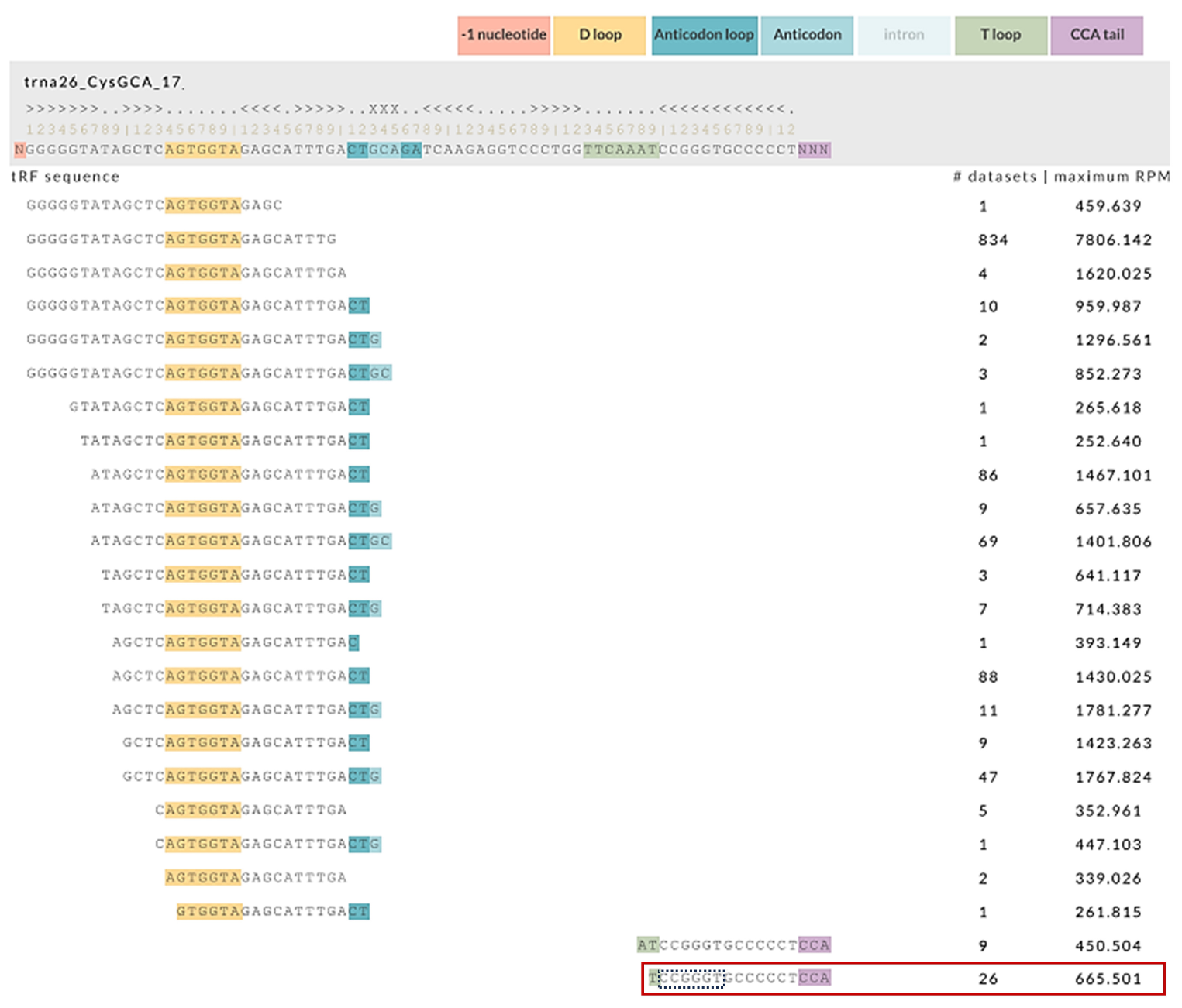


**Fig. S1.** Alignment of 3′-tRF-Cys^GCA^ with the *TRC-GCA2-4* (tRNA^CysGCA^) gene. As shown, it is the most abundant 3′-tRF deriving from tRNAs bearing the GCA cysteine anticodon. The seed region of 3′-tRF-Cys^GCA^ is marked with a blue dashed box.

**
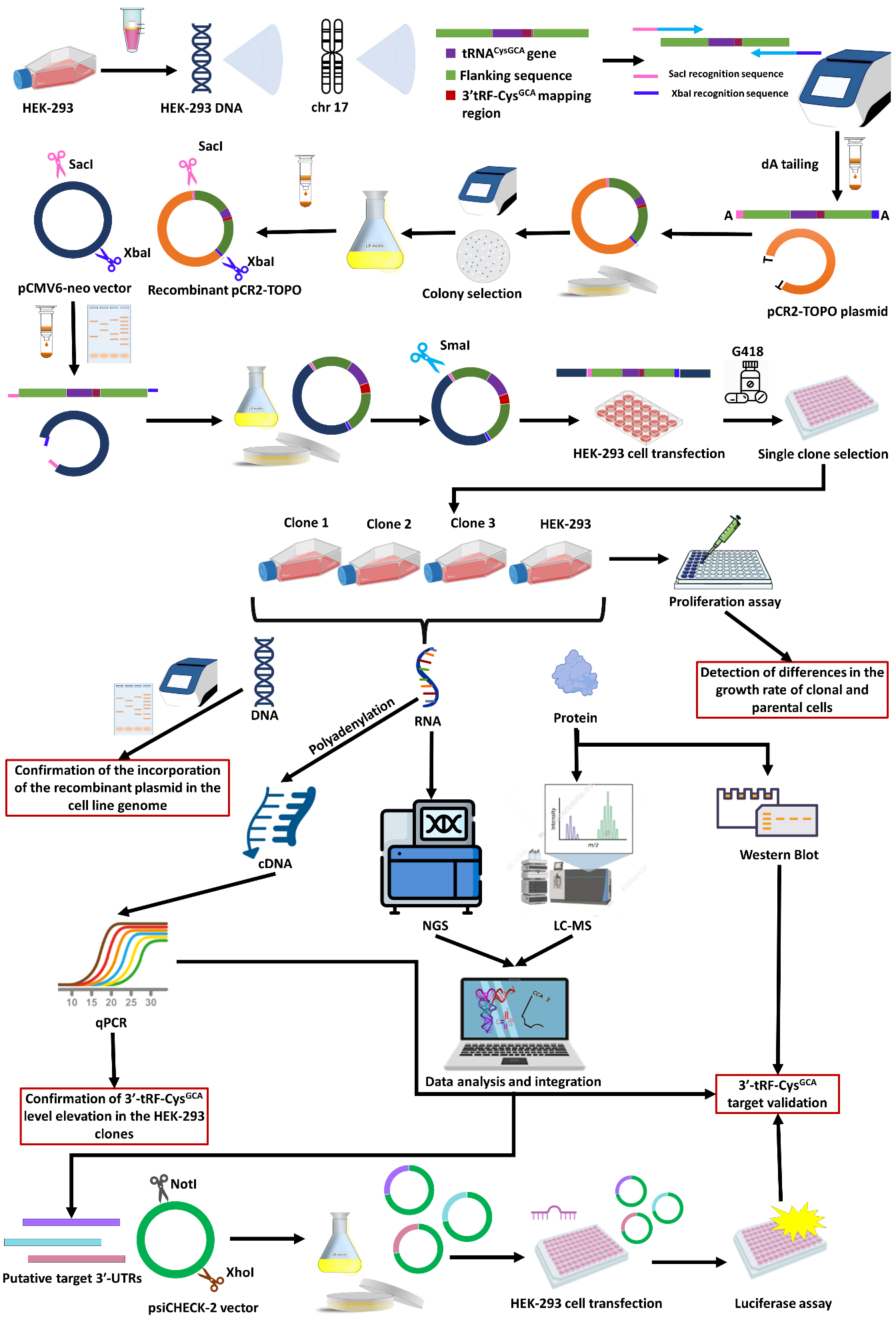
**

**Fig. S2.** Illustration of our experimental workflow for the investigation of the role of 3′-tRF-Cys^GCA^ in the regulation of gene expression.





**Fig. S3.** Alignment of 3′-tRF-Cys^GCA^ with the 3′-UTRs of *TMPO* transcript variant 1 (Refseq ID: NM_003276.2), *ERGIC1* (Refseq ID: NM_001031711.3)*,* and *FTO* (Refseq ID: NM_001080432.3). The complementarity within the seed region of the 3′-tRF-Cys^GCA^ is marked with a red box.


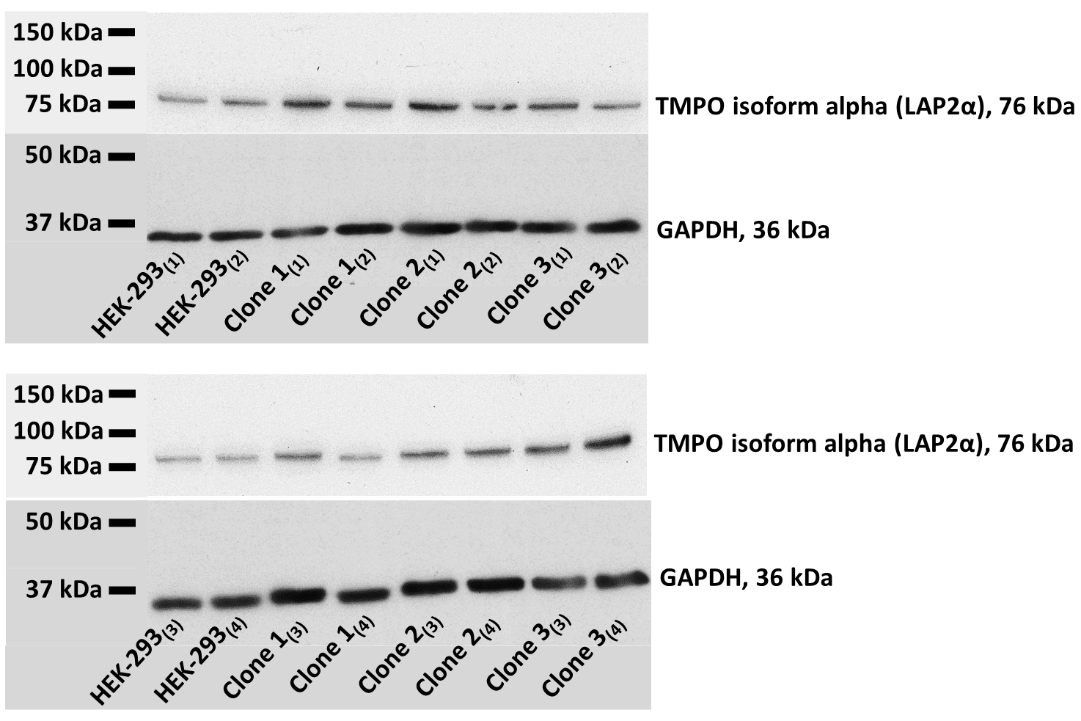


**Fig. S4.** Western blots of TMPO isoform alpha (LAP2α) and GAPDH, in protein extracts of clonal and parental HEK-293 cell lines. Four replicates have been used.
