# Supplementary material for: 3′-tRF-CysGCA overexpression in HEK-293 cells alters the global expression profile and modulates cellular processes and pathways
Source: Funct Integr Genomics. 2023 Nov 21;23(4):341. doi: 10.1007/s10142-023-01272-0 (PMC10663186; doi:10.1007/s10142-023-01272-0)
Supplement: Supplementary file 1 — Supplementary file1 (ZIP 7237 KB) [file 10142_2023_1272_MOESM1_ESM.zip › Supplementary Material/Supplementary Methodology.docx]

**Supplementary Methodology**

**Primer designing and PCR**

Forty (40) ng from the total DNA isolated from the HEK-293 cell line was used as a template for PCR, using KAPA HiFi HotStart ReadyMix PCR Kit (KAPA Biosystems Inc., Woburn, MA, USA) in a MiniAmp Thermal Cycler (Applied Biosystems™, Thermo Fisher Scientific Inc., Waltham, MA, USA). This polymerase ensures the high fidelity of PCR products. The reaction mixture incorporated 12.5 μL 2x KAPA HiFi Hotstard ready mix, 400nM of each primer, and 9.8 μL H_2_O, in a final reaction volume of 25 μL. Regarding the thermal protocol, it was conducted following the manufacturers’ guidelines, with the cycling step being carried out for 35 cycles, while the elongation step was performed at 72ºC for 15s in a MiniAmp Thermal Cycler (Applied Biosystems^™^). The primer annealing temperature is shown in Table S1.

**Plasmid construction, bacteria cell transformation, and plasmid purification**

Five hundred (500) ng of the recombinant pCR™II-TOPO™ vector and the PCMV6-Neo backbone (OriGene, Rockville, MD, USA) were digested using 10 U of each of the SacI and XbaI restriction enzymes (New England Biolabs Ltd., Hitchin, UK) and Cutsmart Buffer. The mixture was heated at 37^o^C for 16h, followed by a heat-inactivation step at 65^o^C for 20 min. The restricted products were loaded in 0.8% agarose gel and both the linearized PCMV6-Neo backbone and the insert for the recombinant pCR™II-TOPO™ vector were cleaned-up using spin columns (Macherey-Nagel GmbH & Co. KG). The procedure was repeated to ligate the linearized PCMV6-Neo backbone and the tRNA^CysGCA^ gene PCR product. After construction and clean-up of the recombinant PCMV6-Neo vector, 10U of the SmaI restriction enzyme (New England Biolabs Ltd.) were used to linearize 1 μg of the recombinant PCMV6-Neo vector, after incubation at 25^o^C for 20h and heat inactivation at 65^o^C for 20 min in Cutsmart buffer.

**Nucleic acid extraction and PCR**

Forty (40) ng of each DNA extract were used as a template to conduct a PCR assay, to ensure the incorporation of the vector in the HEK-293 clone genome. For this purpose, KAPA Taq DNA polymerase (KAPA Biosystems Inc.) was used; the reaction mixture was composed of 18.9 μL nuclease-free H2O, 2.5 μL 10x KAPA Taq Buffer, 400 nM of each dNTP, 400 nM of each primer and 0.5 Units of KAPA Taq DNA Polymerase. The thermal protocol was conducted following the manufacturers’ guidelines, with the cycling step being carried out for 35 cycles, while the elongation step was conducted at 72ºC for 30s in a MiniAmp Thermal Cycler (Applied Biosystems™). The primer annealing temperature is shown in Table S1.

***in vitro* polyadenylation, cDNA synthesis, and real-time quantitative PCR (qPCR)**

Real-time qPCR assays were developed using the KAPA SYBR FAST qPCR Master Mix (2X) Kit (KAPA Biosystems Inc). The reaction mixture included 2.5 μL Η_2_Ο, 1X KAPA SYBR FAST qPCR Master Mix (KAPA Biosystems Inc), 200 nM of each primer, and 0.5 μL cDNA in a final reaction volume of 10μL. The thermal protocol was conducted in a QuantStudio^™^ 5 Real-Time PCR System (Applied Biosystems^™^) and consisted of an initial denaturation step at 95^o^C, followed by 40 cycles of a denaturation step at 95^o^C for 3s and either a coupled annealing and extension step at 60^o^C for 30 s or an annealing step at 64^o^C for 20s and an extension step at 60^o^C for 20s. Moreover, a melt curve was generated after the amplification. A standard curve was generated for each amplicon, using serial cDNA dilutions. A graph was built by plotting the threshold cycle (C_T_) versus the cDNA quantity.

**Quantitative proteomics using data-independent acquisition (DIA)**

The tryptic peptides were cleaned up according to the Sp3 strategy, using a 1:1 mix of Sera-Mag™ SpeedBead Carboxylate-Modified [E3] and [E7] Magnetic Particles (Cytiva, Marlborough, MA, USA). In brief, the beads and the peptides were incubated for 30 min in a 98% acetonitrile solution. The peptides on beads were subjected to two washing steps with 100% acetonitrile and finally eluted from the paramagnetic beads using water with 0.1% formic acid.

The gradient elution (a step of LC-MS/MS analysis) was performed in a UltiMate™ 3000 RSLCnano system (Thermo Scientific™, Thermo Fisher Scientific Inc., Waltham, MA, USA) during a 1 h run, starting with a gradient of 7% Buffer B [0.1% formic acid (v/v) in 80% acetonitrile] to 35% for 40 min, followed by an increase to 45% for 5 min, and a second increase to 99% for 0.5 min, and then kept constant for equilibration for 14.5 min] with a flowrate of 250 nL/min in the main part of the run.

The Q Exactive™ HF-X Mass Spectrometer (Thermo Scientific™) was operated in data-independent acquisition (DIA) mode, in the scan range of 375-1400 m/z using 120,000 resolving power with an automated gain control (AGC) of 3 × 10^6^ and max IT of 60 ms, followed by data-independent analysis, using 8 Th windows (39 loop counts) with 15,000 resolving power with an AGC of 3 × 10^5^ and max IT of 22 ms and normalized collision energy of 26 eV.

**Western blot**

The protein concentration for each protein extract deriving from clonal and parental cells was determined by Bradford assay. Four protein extracts from each cell line were used; 20 μg of each protein extract were run along with a prestained protein marker on a sodium dodecyl-sulfate (SDS) 10% polyacrylamide gel and electroblotted using wet transfer to a nitrocellulose membrane. Equal loading of the samples was confirmed after Ponceau S staining of the nitrocellulose membrane. In each instance, we initiated blocking by utilizing a 5% non-fat dried milk solution in a Tris-buffered saline, 0.1% Tween® 20 detergent (TBST) solution. After this 2-hour blocking stage, the nitrocellulose membrane was rinsed 3 times with TBST solution and horizontally cut allow incubation of each membrane half with a distinct primary antibody, as glyceraldehyde-3-phosphate dehydrogenase (GAPDH) was used as reference.

Two primary antibodies were added at a 1:1000 dilution: a mouse monoclonal anti-LAP2α (Cat. No.: 5369; Cell Signaling Technologies, Danvers, MA, USA) and a horseradish peroxidase (HRP)-conjugated monoclonal anti-GAPDH (Cat. No.: HRP-60004; Proteintech Group Inc., Rosemont, IL, USA). The incubation in the presence of anti-LAP2α was conducted overnight at 4˚C, whereas anti-GAPDH was washed out after a 2-hour incubation period at room temperature. Both membrane halves were rinsed 3 times with TBST solution. Then, a secondary HRP-conjugated goat anti-mouse IgG (Cat. No.: sc-2005; Santa Cruz Biotechnology Inc., Dallas, TX, USA) was added for a 2-hour incubation at room temperature to the membrane half previously incubated with anti-LAP2α, before a final rinse (3 times) with TBST solution. Detection of each targeted protein [TMPO isoform alpha (LAP2α) and GAPDH] was performed by the Enhanced Chemiluminescence (ECL) detection system. Subsequently, the X-ray films were scanned and image analysis was conducted using Quantity One 1-D Analysis Software (Bio-Rad Laboratories, Hercules, CA, USA).
