# Supplementary material for: 3′-tRF-CysGCA overexpression in HEK-293 cells alters the global expression profile and modulates cellular processes and pathways
Source: Funct Integr Genomics. 2023 Nov 21;23(4):341. doi: 10.1007/s10142-023-01272-0 (PMC10663186; doi:10.1007/s10142-023-01272-0)
Supplement: Supplementary file 1 — Supplementary file1 (ZIP 7237 KB) [file 10142_2023_1272_MOESM1_ESM.zip › Supplementary Material/Supplementary Tables/Table S1.docx]

**Table S1.** The primer pairs used in the PCR and quantitative PCR (qPCR) assays.

|  | **Amplified target** | **Sequence (5΄→3΄)** | **Annealing temperature (^o^C)** |
| --- | --- | --- | --- |
| **PCR** | *TRC-GCA2-4* (tRNA^CysGCA^) gene and its flanking sequences | AGGGAGCTCCCAAGATTCCTTAATCTAGTTGGTT | 65 |
|  |  | AGCAGTCTAGACTCACTCGCATTGCATTCTAC |  |
|  | Recombinant plasmid | CAACGGGACTTTCCAAAATGTCG | 60 |
|  |  | GTTCTTCCTTCATAGCCACGCT |  |
| **qPCR** | 3′-tRF-Cys^GCA^ | TCCGGGTGCCCCCTCCA | 64 |
|  |  | GCGAGCACAGAATTAATACGACTCACTATAGG |  |
|  | *SNORD43* | ACTTATTGACGGGCGGACA | 60 |
|  |  | GCGAGCACAGAATTAATACGAC |  |
|  | *SNORD61* | TTGTCCTCTAAGAAGTTCTGAGCTT |  |
|  |  | GCGAGCACAGAATTAATACGAC |  |
|  | *TMPO* transcript variant 1 (*LAP2α*) | ACTGAGGGAACAAGGAACAGA |  |
|  |  | GAGGGAGTAGTTCCAAGTTCAGA |  |
|  | *ERGIC1* | GCTCCCTTATCTGACCCCCA |  |
|  |  | CCCGAAATAAGCAGCCATGC |  |
|  | *FTO* | GGCACGATGCCCTTTGACTT |  |
|  |  | TGAGCTCCGAGAGGAAGAGG |  |
|  | *HPRT1* | TGGAAAGGGTGTTTATTCCTCAT |  |
|  |  | ATGTAATCCAGCAGGTCAGCAA |  |
|  | *B2M* | ACTGAATTCACCCCCACTGA |  |
|  |  | AAGCAAGCAAGCAGAATTTGGA |  |
