# Supplementary material for: 3′-tRF-CysGCA overexpression in HEK-293 cells alters the global expression profile and modulates cellular processes and pathways
Source: Funct Integr Genomics. 2023 Nov 21;23(4):341. doi: 10.1007/s10142-023-01272-0 (PMC10663186; doi:10.1007/s10142-023-01272-0)
Supplement: Supplementary file 1 — Supplementary file1 (ZIP 7237 KB) [file 10142_2023_1272_MOESM1_ESM.zip › Supplementary Material/Supplementary Tables/Table S2.docx]

**Table S2.** Metrics of the next-generation sequencing (NGS) experiment.

| **Cell line** | **Sample number** | **Total reads** | **Aligned reads (%)** | **Average read length (nt^1^)** | **GC content (%)** |
| --- | --- | --- | --- | --- | --- |
| HEK-293 clone 1 | 1 | 14,585,801 | 91.85 | 99.95 | 51.07 |
|  | 2 | 13,371,976 | 90.41 | 99.94 | 53.30 |
| HEK-293 clone 2 | 1 | 14,300,818 | 84.96 | 99.93 | 47.29 |
|  | 2 | 9,609,153 | 90.77 | 99.90 | 48.26 |
| HEK-293 clone 3 | 1 | 16,797,285 | 80.63 | 99.91 | 47.27 |
|  | 2 | 19,048,022 | 96.72 | 99.91 | 48.08 |
| HEK-293 | 1 | 16,521,011 | 97.39 | 99.95 | 48.19 |
|  | 2 | 10,514,671 | 96.57 | 99.92 | 48.28 |
|  | 3 | 8,767,283 | 96.65 | 99.92 | 47.22 |

^1^ nt: nucleotides.
