# Supplementary material for: 3′-tRF-CysGCA overexpression in HEK-293 cells alters the global expression profile and modulates cellular processes and pathways
Source: Funct Integr Genomics. 2023 Nov 21;23(4):341. doi: 10.1007/s10142-023-01272-0 (PMC10663186; doi:10.1007/s10142-023-01272-0)
Supplement: Supplementary file 1 — Supplementary file1 (ZIP 7237 KB) [file 10142_2023_1272_MOESM1_ESM.zip › Supplementary Material/Supplementary Tables/Table S3.docx]

**Table S3.** Sequences cloned in the psiCHECK-2 vector, corresponding to part of the 3′-untranslated regions (3′-UTRs) of the putative 3′-tRF-Cys^GCA^ targets.

| **mRNA** | **Cloned partial sequence of the 3′-UTR (5′🡪3′)** |
| --- | --- |
| *TMPO transcript variant 1* | TTTCTGTTAAGGTTGTTTTAGTTTCCAGATAGGGCTAATTACAAAATGTTAAGCTTCTAC  CCATCAAATTACAGTATAAAAGTAATTGCCTGTGTAGAACTACTTGTCTTTTCTAAAGAT  TTGCGTAGATAGGAAGCCTGGTACAAACAATTTAACGCTT |
| *ERGIC1* | CAGCTTTGGGACCAGGCTGCCCAAAGGTACTCCTTTATACACCCGGCACCTTCCACGAAA  GATGGTACTTCCCAAGCAAGCCCCTATGATTTGTCACTATAGATGGAACCCTGACTTCTG  CCCCATCCCTTCCTGCCCAACCTAGAACCCAGGCCTCAAG |
| *FTO* | AAGGAGCACAAGTCTCAGGCGGAGGAGAAAAAGAGATCGGCTTTTCTCCTCCAACGTTGT  CATGGGCTTAAGCAAGAGCAGTGGAGACTTCTCTTGGCCCCTAGATTGTAGCACCCGGGT  CCCAATCCAAAACAGCTAGGAAATGGTGCCCATGAAGTTT |
