# Supplementary figures and images for: 3′-tRF-CysGCA overexpression in HEK-293 cells alters the global expression profile and modulates cellular processes and pathways
Source: Funct Integr Genomics. 2023 Nov 21;23(4):341. doi: 10.1007/s10142-023-01272-0 (PMC10663186; doi:10.1007/s10142-023-01272-0)

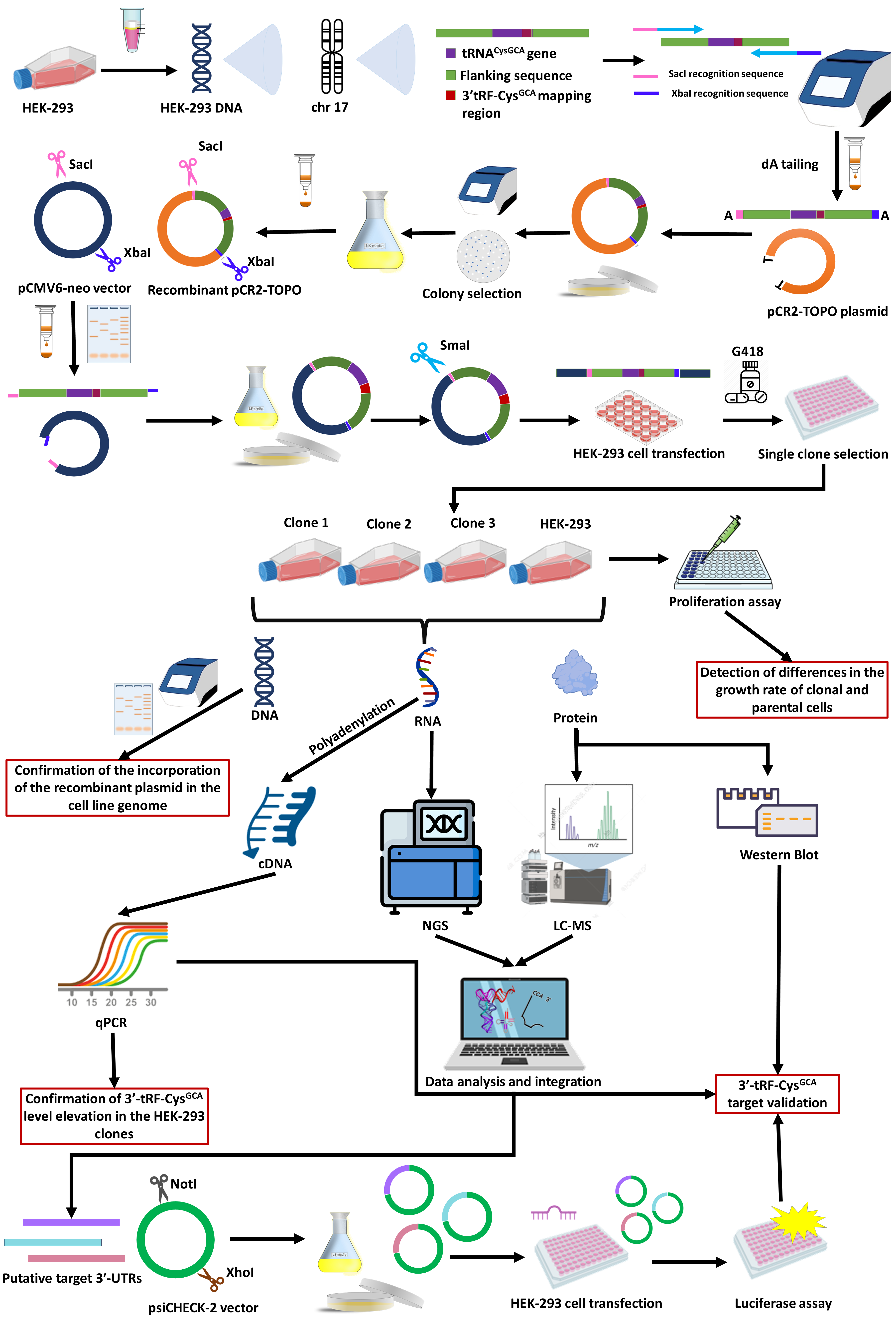

Supplement: Supplementary file 1 — Supplementary file1 (ZIP 7237 KB) [file 10142_2023_1272_MOESM1_ESM.zip › Supplementary Material/Supplementary Figures/Fig S2.tif]

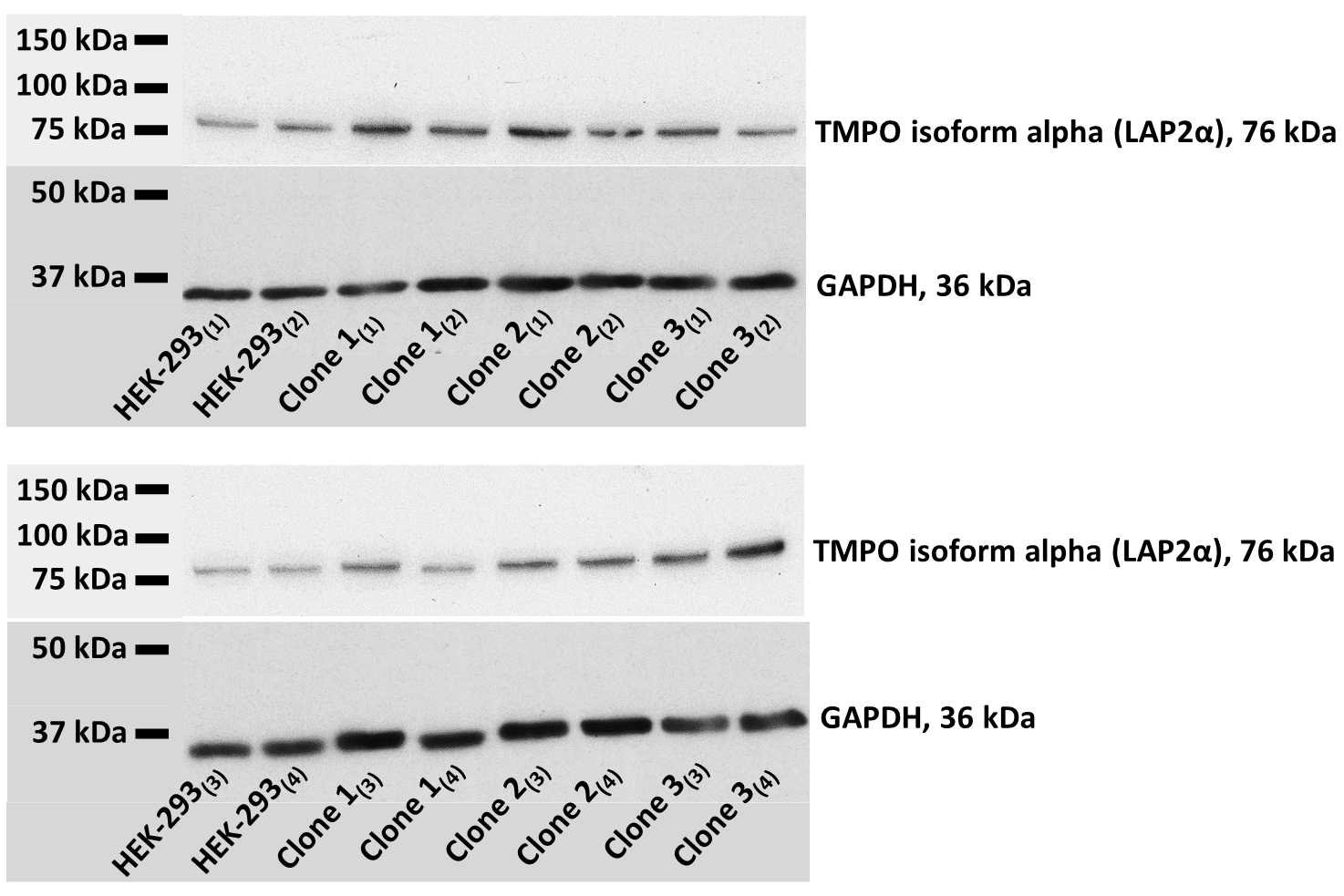

Supplement: Supplementary file 1 — Supplementary file1 (ZIP 7237 KB) [file 10142_2023_1272_MOESM1_ESM.zip › Supplementary Material/Supplementary Figures/Fig S4.tif]
